# Checkpoint blockade therapy in Hodgkin lymphoma: improved response through combination with JAK inhibition

**DOI:** 10.1038/s41392-024-01968-0

**Published:** 2024-09-23

**Authors:** Marc A. Weniger, Ralf Küppers

**Affiliations:** Institute of Cell Biology (Cancer Research), Medical Faculty, University of Duisburg-Essen, Essen, Germany

**Keywords:** Translational research, Molecular medicine

In a recently published article in *Science*, Zak et al. discovered that the Janus kinase (JAK) inhibitor ruxolitinib alters innate and adaptive immune cells to an unanticipated, predominantly immune-stimulatory phenotype in mouse infection models.^1^ This finding motivated a clinical study, which showed that ruxolitinib treatment in combination with the anti-PD1 immune checkpoint inhibitor (ICI) nivolumab in a cohort of relapsed or refractory Hodgkin lymphoma (HL) patients demonstrated promising responses.

The JAK/STAT pathway is a major regulatory pathway in immune responses. Four JAK proteins transmit signals from numerous cytokine receptors in virtually all immune cells to a group of transcription factors, the signal transducers and activators of transcription (STAT). JAK/STAT activity regulates many physiological responses, including cell activation, proliferation, and inflammation. Inhibition of JAK/STAT activity typically has immunosuppressive effects on target cells and immune reactions. Interference of JAK/STAT activity has been investigated for the treatment of many diseases, with the aim to dampen immune responses, leading to approval of the JAK1/JAK2 inhibitor ruxolitinib.

Normal immune responses are tightly controlled. Potentially harmful cells, such as NK cells and cytotoxic CD8 T cells, upregulate inhibitory receptors, including programmed cell death 1 (PD1), during their activation. Different immune cells, e.g., myeloid-derived suppressor cells (MDSC), express the PD1 ligands PD-L1 and PD-L2 that inhibit PD1^+^ target cells to prevent their excessive activity and induce in them an exhausted state. Cancer cells can also acquire expression of PD1 ligands to inhibit antitumor cell attacks. ICIs reactivate immune cells to attack and kill tumor cells and achieved remarkable clinical responses in various cancer types, including melanoma and HL.^2^

Zak and colleagues first systematically screened a chronic infection mouse model for compounds that are able to rescue cytotoxic T cells from exhaustion.^1^ These drugs may potentially be combined and synergize with the effects of ICIs, namely prolong the reactivation and prevent the suppression and exhaustion of antitumor cells. Unexpectedly, the top hits of the screen included several JAK inhibitors, including ruxolitinib. The authors comprehensively characterized the effects of ruxolitinib with and without nivolumab in mouse cancer models. The combination of ruxolitinib plus ICI led to an increase in B, T, and NK cells, of which the latter are essential for the antitumor mechanism. Changes in myeloid-derived cells included a decrease in granulocytes and the reprogramming of granulocytic cells: a reduced expression of suppressive markers, while expression of MHC molecules increased in some of these populations.

Given the successful clinical efficacy of ICIs in patients with HL, the authors then translated their murine findings into a clinical application for HL. HL is a B cell-derived malignancy and one of the most frequent lymphomas. The malignant Hodgkin and Reed-Sternberg (HRS) cells typically account for only about 1% of cells in the tumor tissue, and they are embedded in an inflammatory infiltrate of many different immune cells, including mainly CD4^+^ helper and regulatory T cells, and varying proportions of B lymphocytes, cytotoxic T cells, NK cells, granulocytes, and macrophages.^2^ HRS cells show multiple immune evasion strategies,^3^ but treatment of HL patients with ICIs such as nivolumab has demonstrated excellent clinical responses.^4^ However, some patients fail to respond to ICI therapy or develop resistance. Zak and colleagues assessed the antitumor responses of ruxolitinib combined with nivolumab in a clinical phase I study with HL patients who failed previous ICI therapy. For 19 patients with the combined treatment, an overall response rate of 54% was achieved, and six patients showed a complete metabolic response.^1^ Dose-limiting toxicities were not observed, and adverse events were infrequent. Autoimmune toxicities of grades 1–3 were seen in several patients, so this should be closely followed in future combination studies. These are very promising results.

**Fig. 1:**
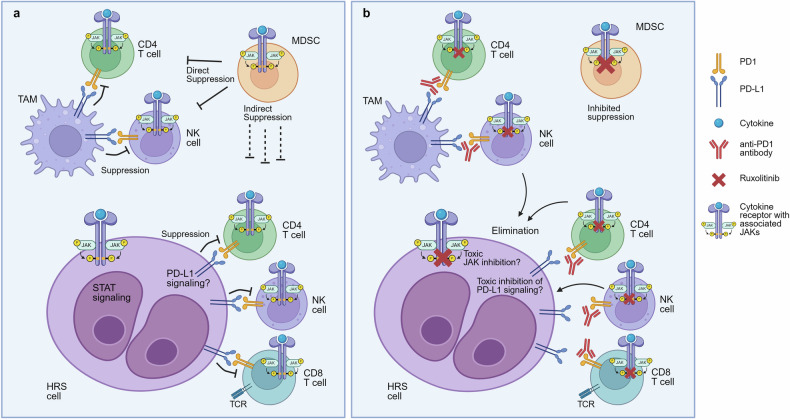
The tumor microenvironment in classical HL and proposed effects of combined JAK and PD1 inhibition. **a** HL tumor microenvironment: suppressed state of effector cells. HRS cells attract various immune cells into the tumor tissue. Effector CD8 T cells, NK cells, and (cytotoxic) CD4 T cells, which potentially could eliminate HRS cells, are silenced by various mechanisms, including PD-L1 expression by HRS cells, tumor-associated macrophages (TAM), and also MDSC. MDSC suppress these effector cells also by additional means. CD8 T cells are mostly incapable of attacking HRS cells, as these, in the majority of cases, have lost MHC class I expression, which is essential for CD8 T cells to recognize their targets with their T cell receptor (TCR). STAT signaling is essential for the survival of HRS cells, and there is an indication that reverse signaling through PD-L1 also supports the survival of HRS cells. **b** Proposed effects of combined JAK1/2 and PD1/PD-L1 inhibition. Inhibition of JAK blocks signaling and alters gene expression programs in various infiltrating immune cells, including MDSC, resulting in inhibited suppressor activity. JAK inhibition may also directly kill HRS cells. It seems that inhibition of effector cell functions is less affected by JAK inhibition. The addition of an anti-PD1 antibody to JAKi blocks the PD-L1/PD1 axis, reactivating antitumor cell effector cells and the elimination of HRS cells. It is not yet clear which effector cells are mainly responsible for the elimination of the HRS cells. Inhibition of PD-L1/PD1 signaling may also contribute to HRS cell killing by impairing reverse signaling through PD-L1 into the HRS cells. Generated with BioRender

A detailed analysis of the peripheral blood mononuclear cells of the treated HL patients revealed an increased fraction of lymphocytes upon combined treatment with nivolumab and ruxolitinib, with more active T cells, a decrease of MDSC and a reduced expression of MDSC markers. These are similar effects as seen in the mouse models initially studied, and they indicate that also in HL, the suppressive effects of JAK/STAT inhibition on MDSC and other suppressive cells overweighs the unwanted inhibitory effect on cytotoxic cells (Fig. 1). However, a caveat of this conclusion is that these analyses could only be done with peripheral blood of the patients. Hence, it remains unclear whether the changes observed upon ruxolitinib treatment are similarly happening in the HL lymph nodes where the elimination of the HRS cells occurs. A further highly relevant question is whether an effect of ruxolitinib on HRS cells directly contributes to its clinical efficacy. Constitutive activity of the JAK/STAT pathway is indeed a hallmark of HRS cells, contributing to their survival and proliferation.^3^ Even though initial clinical trials with JAK inhibitors alone for HL patients did not show promising results, it may well be that in the combination therapy with the ICIs, the direct toxic effect of ruxolitinib on HRS cells contributes to the success of this therapy. A third area which requires further work relates to the mechanisms of anti-PD1 treatment in HL. Whereas the main mechanism of this treatment in various other cancers is the reactivation of suppressed and exhausted cytotoxic CD8 T cells, this does not seem to be the case in HL.^4^ HRS cells in most cases of HL have lost expression of MHC class I, preventing their recognition by CD8 T cells.^5^ It is still unresolved whether the effects of anti-PD1 antibody treatment are mediated by effects on NK cells or on cytotoxic CD4^+^ T cells, or through a reverse, HRS cell-supporting signaling of PD-L1 into the HRS cells, or a combination of these factors. Further studies with the combined treatment of HL patients with ICIs and JAK inhibitors may help to resolve this puzzle.

In conclusion, the elegant study by Zak and colleagues revealed - opposed to initial expectations - that addition of a JAK inhibitor to an ICI treatment of HL patients is not detrimental, but indeed improves therapy outcome. The reason for this is most likely that an unwanted inhibitory effect of JAK/STAT pathway inhibition on cytotoxic immune cells is less severe than expected and overweighed by the inhibition and remodeling of suppressive immune cells.
